# Seroprevalence of brucellosis among cattle slaughtered in three municipal abattoirs of Gombe state, Northeastern Nigeria

**DOI:** 10.14202/vetworld.2016.1082-1086

**Published:** 2016-10-17

**Authors:** Saleh Mohammed Jajere, Naphtali Nayamanda Atsanda, Asinamai Athliamai Bitrus, Tasiu Mallam Hamisu, Ajurojo Oluwaseun Ayo

**Affiliations:** 1 Department of Pathology and Microbiology, Faculty of Veterinary Medicine, Universiti Putra Malaysia, 43400 UPM Serdang, Selangor, Malaysia; 2 Department of Veterinary Public Health and Preventive Medicine, Faculty of Veterinary Medicine, University of Maiduguri, P.M.B 1069 Maiduguri, Borno state, Nigeria; 3 Department of Veterinary Microbiology and Parasitology, Faculty of Veterinary Medicine, University of Maiduguri, P.M.B 1069 Maiduguri, Borno state, Nigeria

**Keywords:** bovine brucellosis, Gombe state, microtiter agglutination test, Nigeria, rose bengal plate test, serum agglutination test

## Abstract

**Aim:**

A cross-sectional study was conducted to determine the seroprevalence of bovine brucellosis among cattle slaughtered at three municipal abattoirs of Gombe State, Nigeria.

**Materials and Methods:**

A total of 200 blood samples collected from slaughtered cattle of different breeds (Sokoto Gudali - 50, White Fulani - 102, Red bororo – 34, and Crossbreeds - 14), sex (males - 19 and females - 181), and from different locations (Billiri - 30, Yamaltu Deba – 50, and Gombe - 120) were screened for brucellosis using rose bengal plate test (RBPT), serum agglutination test (SAT), and microtiter agglutination test (MAT).

**Results:**

Of the 200 serum samples analyzed, 7 (3.5%), 10 (5.0%) and 18 (9.0%) were positive by RBPT, SAT and MAT, respectively. The results showed no statistically significant association between sex and seropositivity to bovine brucellosis. However, seropositivity of bovine brucellosis was higher in females than in males. Similarly, no statistically significant association was observed between breed and occurrence of bovine brucellosis. Moreover, the prevalence of brucellosis was higher in Sokoto Gudali as compared with the other breeds. Based on the study locations, higher seroprevalence was observed in animals screened from Billiri as compared with those from other locations (p<0.05).

**Conclusion:**

The presence of *Brucella abortus* antigen in the sera of slaughtered cattle in Gombe state poses a significant public health risk. Therefore, it is important to carry out further epidemiological studies on fulani herdsmen and cattle herds in the study area, in order to explore the risk factors associated with the occurrence and perpetuation of brucellosis among cattle herds, ascertain the prevalence and status of the disease among both farms and nomadic herds.

## Introduction

Bovine brucellosis is a disease with a significant economic and public health importance due to losses incurred as a result of infertility in animals and extensive chronic morbidity in humans [1]. In Nigeria, bovine brucellosis is a major animal health problem affecting the growth of the cattle industry. It remains a significant disease in animals and humans worldwide and an important cause of reproductive failure such as abortion in cows and sterility in bulls [2-4]. Bovine brucellosis still remains the most widespread form of brucellosis even though reported incidences and prevalence of the disease showed that it varies from country to country. The geographical distribution of the disease is limited. However, it is still a major problem in livestock industry in the Asia, Africa, Latin American, and the Mediterranean regions [5,6].

The disease is transmitted through contamination of feed and water with discharges from infected animals, aerosols, contaminated equipment, fetal fluids, and placental discharges. Infection can also be transmitted via break in the integrity of the skin and mucous membrane [7]. In endemic areas, humans get infected with brucellosis through close contact with infected animals or after birth content of animals, consumption of unpasteurized milk or milk products obtained from either dairy cows, sheep or goats [8,9]. Over the past decades, factors - such as socioeconomic, sanitary, political, and international travel - have greatly impacted on the epidemiology of brucellosis. The occurrence of brucellosis is worldwide, except in Australia, Cyprus, Canada, Norway, New Zealand, Finland, Denmark, and Sweden where the etiologic agent of bovine brucellosis has been eradicated [10].

Bovine brucellosis is responsible for huge economic losses in animal production. This is manifested as abortions, reduced milk yield, and delayed conception; in addition to its zoonotic and public health threat [9]. For instance, an estimated loss of 600 million US dollar due to bovine brucellosis was reported in Latin America. While in the US, an estimated loss due to abortion and reduced milk production was 400 million US dollars [10]. Economic losses due to brucellosis in Nigeria are as a result of abortion, reduced milk production, sterility in bulls, and loss of man-hour in people infected with *Brucella abortus* [11].

Brucellosis is endemic in Nigeria; and the prevalence of bovine brucellosis varies significantly between herds located in the same area, with an estimated seroprevalence in cattle ranging from as low as 0.2% to about 80%. The prevalence of brucellosis in institutional and dairy farms, abattoir surveys and ranches in southern Nigeria ranges from 3.7% to 48.8%. However, the prevalence was low in herd of cattle belonging to the traditional nomadic Fulani than those in the farms and ranches. Similarly, the prevalence of 32.2% was recorded in a herd of cattle belonging to a prison farm. In Northern Nigeria, 25.3% and 19.5% of milk and serum samples were, respectively, reported positive for bovine brucellosis [11].

The occurrence of the disease is influenced by the use of common grazing areas, herding animals of different breeds, age and sex together. Other factors reported to facilitate the occurrence of bovine brucellosis includes, season of the year, lactation, and pregnancy [7,12]. The occurrence of brucellosis in animals has been reported to be a factor limiting the growth and success of livestock in Nigeria [11]. Serological diagnosis of brucellosis in livestock using a well-standardized kit specifically designed for *B. abortus* is considered an important component of disease surveillance and eradication. These tests are also available for use in other livestock other than cattle [13,14]. Despite studies on bovine brucellosis in northeastern Nigerian states such as Yobe, Borno, Bauchi, Adamawa, and Taraba, only a few were conducted in Gombe state and therefore the need for this study.

It is important, therefore, to adopt the use of effective control measures through early and accurate diagnosis of the disease aimed at reducing the prevalence of the disease to boost production. This study was designed to investigate the seroprevalence of bovine brucellosis in three Municipal abattoirs of Gombe state, Northeastern Nigeria.

## Materials and Methods

### Ethical approval

The study to determine the seroprevalence of bovine brucellosis was designed and performed according to the global standard guidelines to care and use of experimental animals described by Ochei and Kolhatkar [15]. Approval was duly obtained from the Institutional Ethics and Research Committee, Faculty of Veterinary Medicine, University of Maiduguri.

### Study area

**Figure-1:**
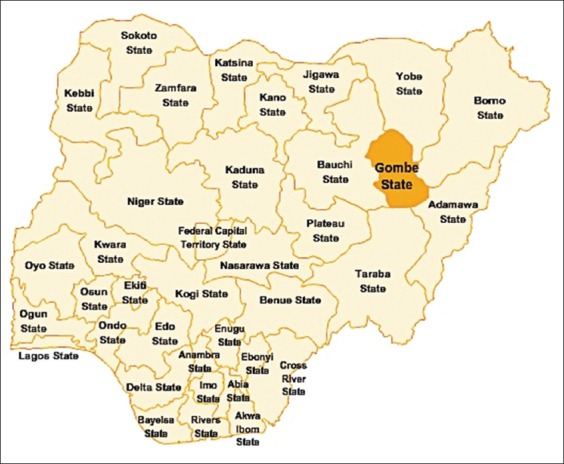
Map of Nigeria showing Gombe state (Source: Google maps).

Gombe state is located in the northeastern geopolitical zone of Nigeria (Figure-1). It shares a common boundary line with Borno, Adamawa, Bauchi, Yobe, and Taraba state along the expansive savannah zone. The state is situated at latitude 9°30’ and 12°30’N and longitudes 8°45’ and 11°45’E. It has an area of 20,265 km^2^ and a population of about 2,353,000 people (NPC, 2006). The state has two different climatic conditions, the dry season ranging from November to March and the rainy season spanning from April to October. It has an average rainfall of 850 mm (https://en.wikipedia.org/wiki/Gombe_State).

### Sample collection

**Figure-2:**
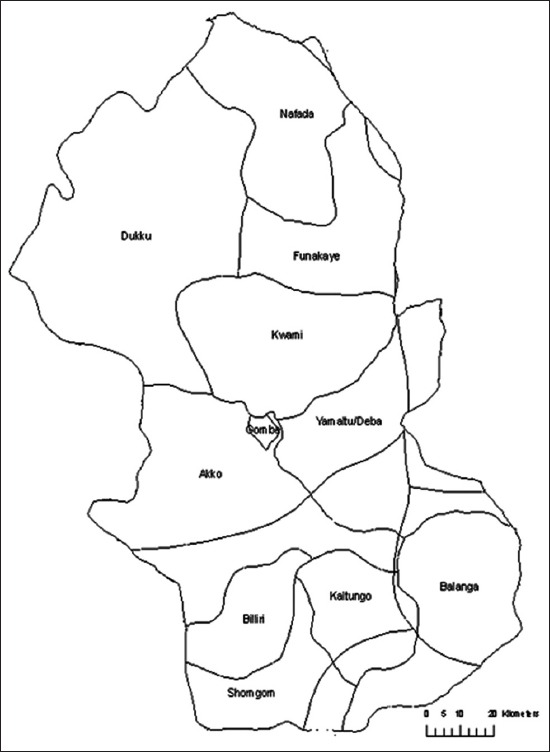
Map of Gombe state showing the three municipal abattoirs.

A total of 200 blood samples were obtained from slaughtered cattle in the municipal abattoirs located in the three agro-ecological zones of Gombe state (Figure-2). Of these, about 91%, 51% and 60% were, respectively, females, males, White fulani breeds and sampled from Gombe metropolis council (Table-1). About 10 mL each of blood was aseptically collected in a well-labeled plain tube without anticoagulant, from the anterior jugular vein of cows slaughtered in these abattoirs. The blood was kept in a transport container and allowed to clot. The sera were collected in 2 mL cryovials after centrifugation at 3000 ×*g* for 5 min and stored at −20°C.

**Table-1:** Baseline characteristics of slaughtered cattle sampled from the three municipal abattoirs in Gombe state, Northeastern Nigeria.

Variables	Categories	Frequency (%)
Sex	Male	19 (9.5)
	Female	181 (90.5)
Breeds	Sokoto Gudali	50 (25)
	White Fulani	102 (51)
	Red Bororo	34 (17)
	Cross breed	14 (7)
Location	Billiri	30 (15)
	Yamaltu Deba	50 (25)
	Gombe	120 (60)
Overall		200 (100)

### Serological reaction

Blood sera was analyzed for *B. abortus* antibodies using rose bengal plate test (RBPT), serum agglutination test (SAT), and microtiter agglutination test (MAT) as described by Adamu *et al*. [7] and Gómez *et al*. [16]. Positive and negative reactions were recorded based on the presence or absence of agglutination reaction. The samples were considered positive for infection with brucellosis if the antigen-antibody titer is three-fold (1.40) or more (one part of the antigen and four parts of the antibody). Antigen for SAT and RBPT standard *B. abortus* antigen Surry KT15 3NB (New Haw Weybridge, UK.) and S99 CVL (Indian Veterinary Research Institute, Izatnagar, Uttar Pradesh, India) were used. The preparation of cocktail was assessed by titration and carried out according to the standard guidelines recommended by the World Organization for Animal Health (OIE) [17]. For SAT, Brucella antigen was prepared in a 1:50 dilution in 0.85% saline solution. A 1:10 to 1:1280 double dilution of serum was carried in tubes containing saline solutions. Appropriate titers of high, low, and negative sera were used as controls. Tubes containing 0.5 ml of diluted serum were mixed with equal amount of 1:50 dilution of Brucella antigen and then incubated at 37°C overnight.

### Statistical analysis

Factors considered to assess their association with seroprevalence of bovine brucellosis include sex, geographical location, and breeds. The results were considered statistically significant at p<0.05. Chi-square test using SPSS version 22.0 (SPSS Chicago, USA), was used to determine the strength of association between the seroprevalence of bovine brucellosis and risk factors.

## Results and Discussion

Bovine brucellosis is considered as one of the most significant bacterial zoonosis hindering the development of the dairy industry in Nigeria. The disease is endemic in many African countries, Asia, Middle East, Central and South America. It is prevalent in areas where effective control programs have not yielded much progress. To this effect, a cross-sectional study was conducted to determine the seroprevalence of bovine brucellosis in three municipal abattoirs in Gombe state. To achieve this, three serological test, namely RBPT, SAT, and MAT were used to screen for cattle sera. The result obtained showed that 7 (3.5%), 10 (5.0%) and 18 (9.0%) of the cattle sera analyzed were positive by RBPT, SAT and MAT, respectively (Table-1). Statistically, no significant difference was observed between the seropositivity of the disease and the use of RBPT and SAT as a diagnostic tool in screening for bovine brucellosis. However, a statistically significant difference was observed between the seropositivity of the disease and the sensitivity of MAT in detecting antibodies to *B. abortus* in the sera screened for bovine brucellosis

The seroprevalence of bovine brucellosis as reported in this study was lower than the seroprevalence of bovine brucellosis reported in Yobe (34.0%), Adamawa (36.6%), and Bauchi state (5.4% and 3.9%) respectively [7]. Similarly, in southern Nigeria, Cadmus *et al*. [18] reported a seroprevalence of 6% in 2004, 6.17% in 2005 and 5.31% in 2006, which was higher than the seroprevalence of bovine brucellosis reported in this study. Furthermore, in another study, Cadmus *et al*. [19] reported a seroprevalence of 8.6% in three cattle production system in southwestern Nigeria. This difference in the seroprevalence of the disease could be attributed to the difference in breeds, sensitivity of test kits, seasonal variation, farming system, and sample size. In addition, lower rates of bovine brucellosis was reported in this study than those reported in other African countries such as Ethiopia (4.9%) [2], Central African Republic (4.9%) [20], Eritrea (4.2%) [21], Chad (7%) [22], Jordan (6.5%) [23], and Cameroon (8.4%) [24]. In Eastern Sudan, Gumaa *et al*. [12] reported seroprevalence of 2.15%, after sampling 2500 serum samples collected from sheep. The difference in prevalence could be due to the difference in breeds, geographical location, sample size, serological techniques, and inter-laboratory variation. Similarly, Junaidu *et al*. [25] also reported a much higher (19.5%) seroprevalence of bovine brucellosis after sampling 1,547 serum samples in Sokoto, Northwestern Nigeria. In another study, Zubairu *et al*. [26] reported a seroprevalence of 21.3% in cattle sera collected in Taraba state, North-eastern Nigeria. This disparity in the seroprevalence of bovine brucellosis in different parts of the country is in congruent with the report of Mai *et al*. [11]. Where the authors reported that the prevalence of bovine brucellosis varies between animals in the same agro-pastoral zone. Even though the seroprevalence of brucellosis reported in this study was lower using RPBT and SAT, it was however higher with MAT (Table-2).

**Table-2:** Seroprevalence of bovine brucellosis among slaughtered cattle in Gombe state, Northeastern Nigeria.

Variables	Number sampled	Number of positive animals (%)		

		RBPT	MAT	SAT
Sex				
Male	19	0 (0)	1 (5.3)	0 (0)
Female	181	7 (3.9)	17 (9.4)	10 (5.5)
Breeds				
Sokoto Gudali	50	3 (6.0)	8 (16.0)	4 (8.0)
White Fulani	102	3 (2.9)	9 (8.8)	5 (4.9)
Red Bororo	34	1 (2.9)	1 (2.9)	1 (2.9)
Cross breeds	14	0 (0)	0 (0)	0 (0)
Locations				
Billiri	30	2 (6.7)	3 (10.0)	4 (13.3)
Yamaltu Deba	50	2 (4.0)	6 (12.0)	3 (6.0)
Gombe	120	3 (2.5)	9 (7.5)	3 (2.5)
Overall	200	7 (3.5)	18 (9.0)	10 (5.0)

RBPT=Rose bengal plate test, MAT=Microtiter agglutination test, SAT=Serum agglutination test

In this study, the seropositivity of bovine brucellosis was higher in Sokoto gudali and White fulani as compared with Red bororo and crossbreeds. However, there was no statistically significant association between breeds and occurrence of brucellosis in cattle (p>0.05) (Table-2). This finding is in congruent with the report of Junaidu *et al*. [24], where the authors reported a higher prevalence of bovine brucellosis in Sokoto Gudali (29.59%) breeds. In addition, even though the proportion of bovine brucellosis was higher in females than in males, the difference was not statistically significant (p>0.05) (Table-2). This is because female cows remain the locale of infection, which helps to spread the disease from one animal to another, either through lactation or during mating. Pregnancy and lactation were reported to enhance susceptibility to infection. The growth of virulent strains of Brucella organism was reported to be stimulated more in females because of the presence of higher volume of D-erythritol normally found fetal tissue than in testes and seminal vesicle [24]. These thoughts provide proofs as to the different rates of seroprevalence of bovine brucellosis observed in this study.

The risk of bovine brucellosis is not only restricted to the animal husbandry alone but also represent significant zoonotic implications characterized by debilitating and severe complications in humans [1]. Serological studies have shown that bovine brucellosis is a common problem in many grazing zone in Nigeria [7]. From reports, it was observed that there was variation in the prevalence of bovine brucellosis in many countries, the surveillance strategies adopted for the control and prevention of the disease is generally very poor. Purchase of infected animals as replacement cattle, interaction with wildlife, free movement of animals by nomads, change in climatic conditions, deforestation, and the system of animal production, regulatory issues and demographic factors were considered as likely factors that increase the spread of bovine brucellosis. Other factors include sharing of bulls between farmers, the practice of free range grazing and movement as a result of trade have greatly increased the risk of exposure to brucellosis in cattle [7].

## Conclusion

The presence of *Brucella abortus* antibodies in the sera of cattle slaughtered in the municipal abattoirs of Gombe state poses a significant public health risk. Therefore, it is imperative to design an effective control and preventive measures aimed at reducing the spread of brucellosis in cattle and subsequent exposure to humans in the study area.

## Authors’ Contributions

Authors SMJ and NNA conceived and designed the study. AAB and TMH contributed in literature review and data analysis. AOA conducted sampling from the abattoir. SMJ and AAB drafted the first manuscript. NNA and TMH revised the manuscript. All authors read and approved the final manuscript.
